# Fas Signalling Promotes Intercellular Communication in T Cells

**DOI:** 10.1371/journal.pone.0035766

**Published:** 2012-04-25

**Authors:** Francesca Luchetti, Barbara Canonico, Marcella Arcangeletti, Michele Guescini, Erica Cesarini, Vilberto Stocchi, Mauro Degli Esposti, Stefano Papa

**Affiliations:** 1 Department of Earth, Life and Environmental Sciences, University of Urbino “Carlo Bo," Urbino, Italy; 2 Department of Biomolecular Sciences, University of Urbino “Carlo Bo," Urbino, Italy; 3 Faculty of Life Sciences, University of Manchester, Manchester, United Kingdom; 4 IIT Genova, Genova, Italy; Mayo Clinic, United States of America

## Abstract

Cell-to-cell communication is a fundamental process for development and maintenance of multicellular organisms. Diverse mechanisms for the exchange of molecular information between cells have been documented, such as the exchange of membrane fragments (trogocytosis), formation of tunneling nanotubes (TNTs) and release of microvesicles (MVs). In this study we assign to Fas signalling a pivotal role for intercellular communication in CD4+ T cells. Binding of membrane-bound FasL to Fas expressing target cells triggers a well-characterized pro-apoptotic signalling cascade. However, our results, pairing up flow cytometric studies with confocal microscopy data, highlight a new *social dimension* for Fas/FasL interactions between CD4+ T cells. Indeed, FasL enhances the formation of cell conjugates (8 fold of increase) in an early time-frame of stimulation (30 min), and this phenomenon appears to be a crucial step to prime intercellular communication. Our findings show that this communication mainly proceeds along a cytosolic material exchange (ratio of exchange >10, calculated as ratio of stimulated cells signal divided by that recorded in control cells) via TNTs and MVs release. In particular, inhibition of TNTs genesis by pharmacological agents (Latruculin A and Nocodazole) markedly reduced this exchange (inhibition percentage: >40% and >50% respectively), suggesting a key role for TNTs in CD4+ T cells communication. Although MVs are present in supernatants from PHA-activated T cells, Fas treatment also leads to a significant increase in the amount of released MVs. In fact, the co-culture performed between MVs and untreated cells highlights a higher presence of MVs in the medium (1.4 fold of increase) and a significant MVs uptake (6 fold of increase) by untreated T lymphocytes. We conclude that Fas signalling induces intercellular communication in CD4+ T cells by different mechanisms that seem to start concomitantly with the main pathway (programmed cell death) promoted by FasL.

## Introduction

Communication with the environment is a basic principle of any biological system. With the increasing complexity of higher organisms, cells had to evolve several different mechanisms to exchange spatial and temporal information crucial for tissue organization and the maintenance of the organism as a whole [1]. Until recently cell-to-cell communication was considered as the interaction between diffusible molecules like hormones and growth factors that bind specific receptors in/on target cells [2]. Recently, new types of cell-to-cell communication have been reported to occur among mammalian cells. In 2003, Hudrisier and Joly [3] first reported that several cell types of the immune system exchange surface elements and membrane fragments.

Several studies have shown that patches containing membrane-anchored proteins and lipids can move from the surface of one cell to that of another following the formation of the immune synapse (IS). This synapse-mediated process has been called “trogocytosis" [3] and allows the transfer of surface molecules that remain functional; consequently their acquisition confers new properties to cells of the immune system, with multiple consequences [4]–[7]. Other reports have demonstrated that trogocytosis, or similar phenomena, can occur through the formation of membrane bridges [8], tunneling nanotubes (TNTs) [9]–[11] and finally through the secretion of vesicles including exosomes [12]. The term trogocytosis has retained the original concept of exchange of membrane fragments, but often encompasses one or more of these different forms of intercellular communication [13].

In 2004, Rustom et al. [14] uncovered a new route of cell-to-cell communication between animal cells, based on the formation of membranous nanotubes bridging rat pheochromocytoma (PC12) cells. These structures, named TNTs, were found to form *de novo* between cells and facilitate the intercellular transfer of vesicles of endocytic origin as well as, on a limited scale, other membrane components and cytoplasmic molecules. Later, evidence was provided that such nanotubular bridges could have important physiological functions in cell-to-cell communication during health and disease. In particular, Sowinski et al. [10] have reported closed ended nanotubes between resting T cells, which can allow the rapid spread of HIV-1 particles [15], [16]. Owing to the lack of a specific marker for TNTs, the identification of these structures has been mainly based on the morphological criteria and the presence of cytoskeletal elements. Conversely, several authors have distinguished two types of TNTs, type I and II. TNTs of type I usually are short not longer then 30 µm and highly dynamic structures containing actin filament, whereas TNTs of type II are much longer (even more than 100 µm), appear to be more stable. Several reports have shown that TNTs of type II contain both F-actin and microtubules and appear to be thicker (>0.7 µm diameter) than type I TNTs [17]; while, according to other authors, they contain instead cytokeratin filaments, which are probably responsible for their stability and longer life span [18]. An alternative, medium to long range way of establishing intercellular communication is *via* the release of membrane vesicles, which have recently attracted increasing interest. Membrane vesicles are complex structures composed of a lipid bilayer that contains transmembrane proteins and enclosed soluble components derived from the cytosol of the donor cell [19]. These vesicles have been shown to affect the physiology of neighbouring recipient cells in various ways, including *via* the exchange mRNA and microRNAs [20], [21]. The presence of nucleic acid in secreted vesicles has raised the exciting possibility that transfer of genetic material might affect the function of recipient cells [22]. Recently intracellular communication has been associated also to a signalling of apoptosis mediated by death receptor Fas.

Fas/Apo-1 (CD95) is a receptor of the TNF family that is expressed at the surface of activated T lymphocytes and is physiologically involved in their peripheral deletion activation-induced cell death (AICD) [23], [24]. The ligand of Fas receptor, FasL or CD95L, is a transmembrane protein that is also associated to secreted vesicles [25]. During primary stimulation of cytotoxic T lymphocytes and NK cells, newly synthesized FasL is directed to and stored in specialized secretory lysosomes. Thus, in activated human T cells, FasL is a transmembrane component of these lytic granules that also contain granzymes and pore-forming perforin [26]. Upon interaction with a target cells, the lipid bilayer of the secretory lysosomes is believed to fuse with the plasma membrane thereby releasing the soluble factors into the IS and presenting FasL on the cell surface. To date, the association of Fas-induced apoptosis to cell-to-cell communication has not been studied in detail, especially in primary lymphocytes. Wang and co-workers [27] have indicated that TNTs might be a mechanism for cells to respond to harmful signals and transfer cellular substances or energy to another cell under stress. Our previous work [28] has shown for the first time a “new role" for Fas signalling demonstrating that Fas stimulation rapidly induces extensive membrane nanotube formation between neighbouring T cells. The aim of this work is to expand the initial observations and elucidate the types of cell-to-cell communication that occur in the early phases of the apoptotic pathway leading to peripheral deletion CD4+ T cells *via* Fas stimulation.

## Results

### Choice of lipophilic probes to evaluate extracellular exchange

To evaluate intercellular exchanges between lymphocytes we have screened different dyes and staining conditions that allowed flow cytometry approaches. We have consistently found that components of the plasma membrane and of the cytoplasm tend to exchange spontaneously between resting T cells, albeit with different basal levels. Besides the probes studied before, we have concentrated this study on four dyes that have been previously used to evaluate intercellular communication. The fundamental properties of these dyes, which are substantially in agreement with the data reported by Daubeuf et al. [29], are shown in Table 1.

**Table 1:** Evaluation of passive diffusion.

	PROPERTIES OF THE PROBE	PEAK ex	PEAK em	CHANNEL	PASSIVE DIFFUSION
**PKH-67**	Green fluorescent probe. Vital lipophilic and membrane intercalating dyes	**490 nm**	**502 nm**	**FL1**	•
**PKH-26**	Red fluorescent probe. Vital lipophilic and membrane intercalating dyes	**551 nm**	**567 nm**	**FL2**	•
**DiI C18**	Red Orange fluorescent probe. Lipophilic tracer (Long chain dialklylcarbocyanines)	**556 nm**	**572 nm**	**FL2**	•
**CFSE**	Cytosolic dye retained in living cells (transformed inside the cell into molecule highly fluorescent and non-permeant to the cell membrane).	**491 nm**	**518 nm**	**FL1**	••

The formula reported in the section 2.3 of materials and methods has been applied to calculate the spontaneous transfer of each dye.

•Corresponds to value <0.2%.

••Correspond to probe that diffuse in the 0.2–0.8% range.

### Fas signalling increases the formation of cell conjugates

First, we investigated the FasL–induced apoptotic pathway by means of Annexin-V (Anx-V) and caspase detection. In particular, apoptotic cells were detected after 30 and 120 min of FasL administration (Fig. S1). The data shown in Fig. S1 indicate that FasL is able to induce significant levels of caspase dependent apoptosis in our cellular settings. We next evaluated intercellular communication associated with FasL treatment, following the guidelines reported in recent literature.

**Figure 1:**
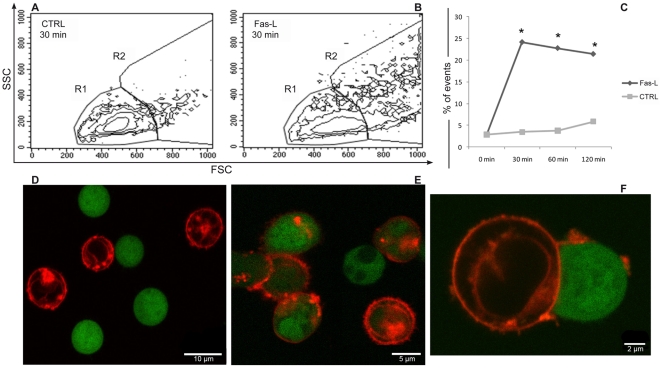
FasL promotes cell conjugates formation in CD4+ T lymphocytes. Contour plots show CD4+ T cells untreated (A) and treated with FasL (B) for 30 min. The treatment increases the percentage of cell conjugates identified by gate R2 drawn on physical parameters of scattering (FSC/SSC). Statistical analysis of CD4+ T cells shows the percentage of cell conjugates at different times with (Fas-L) or without (CTRL) FasL treatment (C). The histogram highlights that FasL administration led a sharp increase of cell conjugates within 30 min, slightly decreasing later. * indicates statistical significance: p<0.05 (n = 6 experiments). The formation of cell conjugates after FasL treatment is also documented by confocal microscopy (D–F). CD4+ T cells were labelled with CFSE (green) and DiI (red). In control condition (D), the cells appear isolated with distinct fluorescences, whereas in the FasL samples (E,F) the cells are tightly linked with mixed fluorescences. These observations attest an intercellular exchange of DiI+ and/or CFSE+ patches.

**Figure 2:**
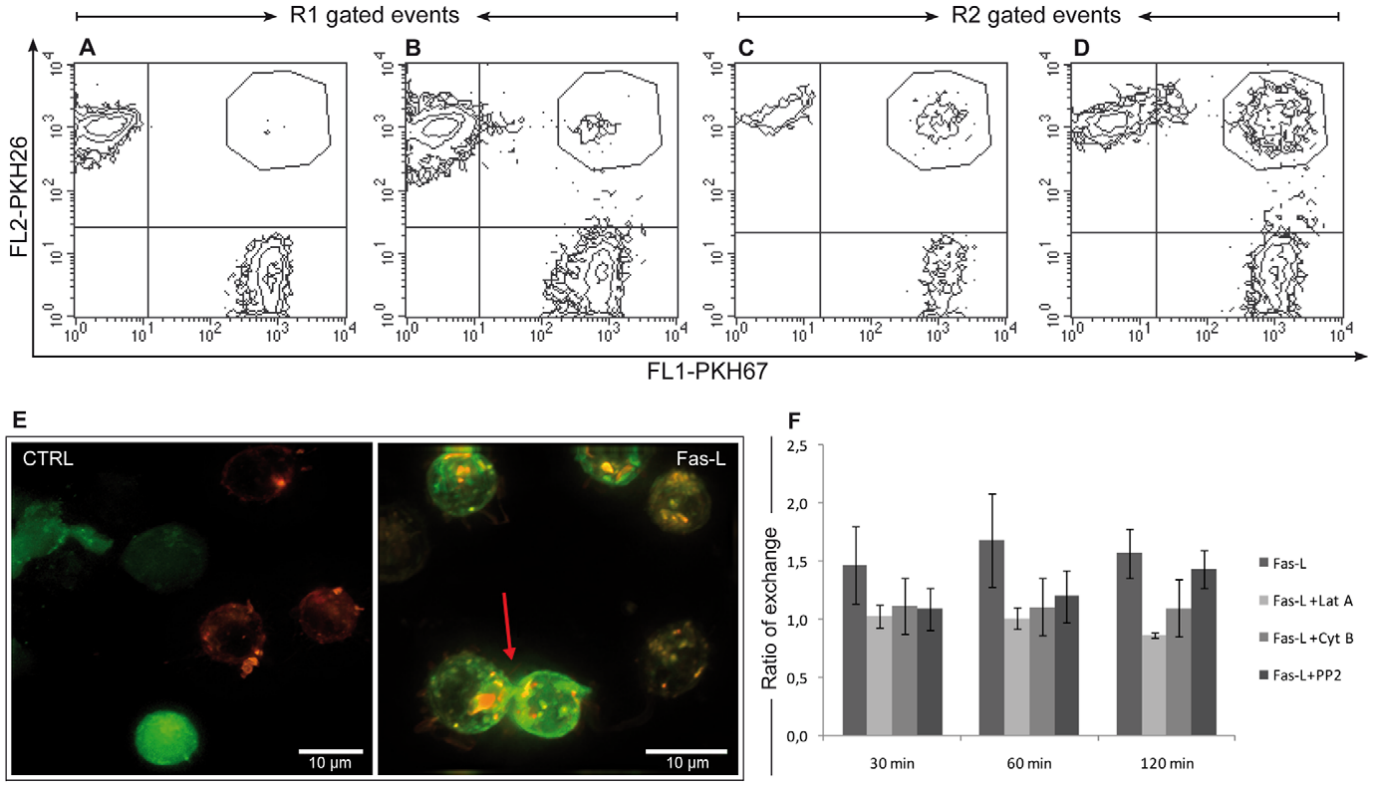
Detection of plasma membrane fragments exchange by PKH26 and PKH67 dyes. PKH26 and PKH67 labelled CD4+ T cells were co-cultured for 30 min without (A,C) or with (B,D) FasL addition. (A,B) represent the events selected by R1 gate (isolated cells) and (C,D) the events selected by R2 gate (cell conjugates) (see Fig. 1). Contour plots highlight an higher double positive events (identified by drawing area) after FasL treatment, in particular within gate R2 than in gate R1. Imaging microscopy (E) shows CD4+ T cells stained with CFSE and DiI at the outset (CTRL) and after 30 min FasL treatment (Fas-L). The tick arrow indicates patches of exchange material between conjugated cells bound also with a nanotube. The PKH67 intercellular exchange (F) was evaluated after FasL treatment with or without different inhibitors, Lat A (Latrunculin A), Cyto B (Cytochalasin B) and PP2. The scatter gram shows a poor membrane transfer (1.5–3 ratio of exchange) which was reduced by pharmacological agents, in particular after Lat A treatment. Results from n = 6 separate experiments between FasL and inhibitors cited above were statistically significant (p<0.05).

Previously Poupout [30], [31] have used stringent gating strategy to remove cell conjugates from the count of cells exhibiting exchange of specific dyes. As we have recently demonstrated [32] is important to evaluate also cell conjugates, because the formation of stable cells conjugated, i.e. detectable by flow cytometry, is a pre-requisite for the cell-to-cell communication and the subsequently exchange of cellular material. The formation of cell conjugates was identified by performing a specific gate strategy to effectively separate cell conjugates as shown in Fig. 1. CD4+ T cells were gated according to their physical appearance using the forward scatter (FSC) and side scatter (SSC) parameters, to exclude debris and late-appearing apoptotic cells (gate R1 in Figs. 1A and B). Purified CD4+ T cells treated with FasL showed a sharp increase in the number of cell conjugates (gate R2 in Fig. 1B). Maximal increase in cell conjugates (from an average of 2.9% of control cells to an average of 24.1% of treated cells) occurred after 30 min of FasL treatment; subsequently, the percentage of cell conjugates decreased slightly (Fig. 1C). The differences in cell conjugates between untreated and the Fas-stimulated samples remained statistically significant (p<0.05) at every time point (Fig. 1C). Our new approach identified for the first time a rapid increase in cell conjugates following Fas stimulation with FasL using flow cytometry. These results were confirmed also by confocal microscopy as shown in Figs. 1E and F. The microscopic images of CD4+ T cells stained with CFSE (carboxyfluorescein diacetate succinimidyl ester) and DiI (1,1′-dioctadecyl-3,3,3′,3′-tetramethylindocarbocyanine perchlorate) also indicated an increase extent of cell conjugates with some exchange of colour after FasL treatment (Figs. 1E and F), in accordance with previous findings [28]. Whereas it is straightforward to distinguish cell conjugates with exchanged material (mixed colours) from cell conjugates exhibiting no exchange material by using fluorescence microscopy, the same is not true when using flow cytometry. Classically cells with mixed colours have been identified as a shift of FL1 or FL2 channel in contour plots (Figs. 2B and D). However, double positive events (upper right quadrant) could also correspond to conjugates of red and green cells exhibiting no exchanged material, particularly when they display the same fluorescence intensity of single red or green cells. These double positive events were particularly evident after FasL treatment (Figs. 2B and D) and when the analysis was performed within gate R2 (Fig. 2D). Given that it is impossible to precisely distinguish these double red/green events between simple conjugates and cells displaying mixed colours (upper right quadrant), we only excluded them during subsequent analysis of cell-to-cell communication. Deconvolution microscopy images confirm the presence of cell conjugates and at the same time the exchange of differentially coloured dyes after FasL treatment (Fig. 2E).

### Pharmacological manipulation of intercellular exchanges stimulated by Fas signalling

Our results obtained with plasma membrane lipophilic dyes (Table 1) indicated that FasL treatment induced, after an early wave of cell conjugation, exchanges of membranes detectable by flow cytometry up to 2 h after FasL treatment (Figs. 2A–D and F). We next investigated how these exchanges could be manipulated by selected pharmacological agents. Inhibitors of actin polymerization or of src kinases have been documented to partially or fully reduce trogocytosis in T cells, while having no effect on the trogocytosis observed in B cells [33]. We next explored how the actin destabilizing agents latrunculin A (2.5 µg/ml) and cytochalasin B (10 µM), in comparison with the general tyrosine kinase inhibitor, PP2 (10 µg/ml) [33], would affect the FasL-enhanced exchanges of green PKH-labelled membrane. These pharmacological agents affected the uptake of each dye in different ways, especially at prolonged times of incubation. Latrunculin A, a specific inhibitor of actin polymerization, was most potent in reducing the exchange of PKH67 (p<0.001), followed by cytochalasin B (p<0.01) and PP2 (p<0.05) (Fig. 2F). It is important to remark that the levels of receptor-enhanced exchanges of membrane fragments are relatively low (1.5–3 ratio of exchange) in comparison with those mediated by other receptors, as shown in a recent work of Hudrisier and Joly [34].

### Evaluation of the exchange of cytoplasmic material

**Figure 3:**
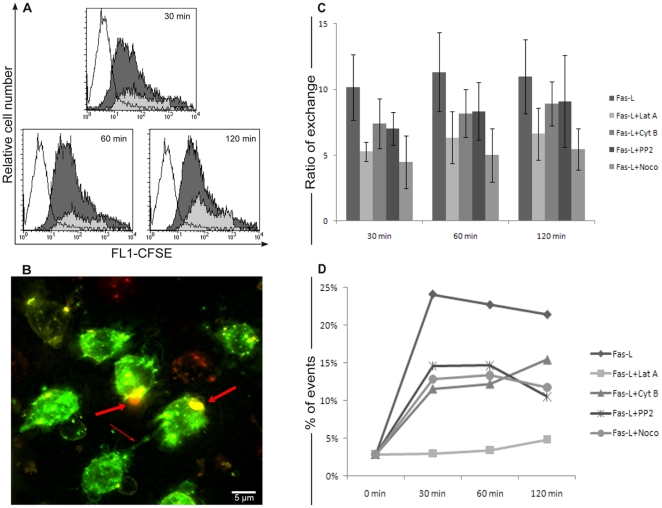
Cytoplasmic transfer evaluation by CFSE stain. Data are presented as an overlay of three different cytometric histograms (A). Empty histograms represent untreated cells at the outset. Grey and light grey histograms are generated using R1 and R2 gates (see Fig. 1) and therefore represent isolated and conjugated cells, respectively. The results show that MFI is higher in R2 gated events if compared to R1 gated events for each time points. CD4+ T cells staining with CFSE and DiI after 30 min of FasL treatment were analyzed by fluorescence deconvolution microscopy (B). The image shows a nanotube connecting two cells (thin arrow) and the uptake of red patches by green cells (thick arrows). Time-course of CFSE exchange (C) was evaluated for the indicated times of FasL treatment with different pharmacological agents, Lat A (Latrunculin A), Cyto B (Cytochalasin B), PP2 and Noco (Nocodazole). In particular, it is possible to observe a significant CFSE uptake from red cells, which was strongly inhibited by Lat A and Noco. Results from n = 6 separate experiments showed statistically significant differences for all inhibitors (p<0.05). Percentage of cell conjugates (D) was compared among all experimental conditions (FasL *vs* specific inhibitors). Statistical analyses of inhibitors effect on CFSE exchange (C) and cell conjugates formation (D) highlight a different behaviour of Lat A and Noco. Lat A treatment led a strong decrease of both phenomenon, whereas Noco treatment affected majorly cytoplasmic material transfer.

Our previous observations indicated that FasL treatment induced the formation of open-ended nanotubes allowing the uptake of cytosolic material from one connected cell to the other [28]. We verified these observations further in CD4+ T cells red-labelled with the lipophilic stain PKH26 and then mixed with cells previously labelled with CFSE in a 1∶1 ratio. CFSE is considered a cytoplasmic dye that penetrates cells in a passive way. Once taken up by cells, its acetate groups are cleaved by intracellular esterases and the resulting carboxyfluorescein then emits intense green fluorescence, a property that has been largely exploited in studies of cell proliferation and viability [35]. The treatment with FasL induced a large transfer of CFSE fluorescence between cells, as shown by the marked shifts in green fluorescence of red-labelled cells (grey histograms in Fig. 3A). The uptake of CFSE by PKH26+ cells (Fig. 3) clearly suggested that Fas stimulation with its cognate ligand, FasL, produces an exchange of cytosolic materials between cells.

Of note, large shifts in green fluorescence (grey histograms in Fig. 3A) were observed in cells selected with both the gating regions R1 and R2 (cf. Fig. 1 and Fig. 2). When we included, as before, the R2 events associated with cell conjugates (in which we excluded FL1/FL2 doublets as explained above), a different shift of green fluorescence was obtained (light grey histograms in Fig. 3A). These results indicate that performing the analysis within the gate R1 or gate R2 leads to a different ratio of exchange, as revealed by the different values of median fluorescence intensity (30 min: 34.4 of R1 *vs* 61.5 of R2; 60 min: 45.3 of R1 *vs* 89 of R2; 120 min: 44 of R1 *vs* 89 of R2). These value were obtained using the formula suggest by Daubeuf and co-workers [29]. This suggested us to maintain cell conjugates detected by scatter plots (cf. Fig. 1A) in subsequent analyses.

The deconvolution cytofluorescence images in Fig. 3B clearly show the presence of nanotubes connecting cells exhibiting an exchange of CFSE-labelled cytoplasmic material, consistent with the results obtained by flow cytometry. These nanotubes, as previously reported [28], could explain the origin of the large CFSE transfer observed by flow cytometry after Fas stimulation. We then studied whether the pharmacological agents we had previously tested on membrane exchanges could affect the nanotube-mediated exchange of cytoplasmic material. There is a considerable heterogeneity between membrane nanotubes. In fact, we found that treating CD4+ T cells with latrunculin A, cytochalasin B, PP2 and nocodazole before FasL addition resulted in significant decrease in the uptake of CFSE (Fig. 3C), which was statistically significant: p<0.05 for latrunculin A, cytochalasin B and PP2 and p<0.001 for nocodazole.

Overall, our data with pharmacological agents indicated that the Fas-enhanced exchange of CFSE (cytosol) depended not only on actin filaments, which constitute the basic structures of membrane nanotubes, but also on the microtubule network, which is specifically disrupted by nocodazole. Interestingly, latrunculin and nocodazole produced a larger effect on cytoplasmic exchange (with 46% and 56% of inhibition, respectively), than PP2 and cytochalasin (≤27%) (Fig. 3C). These results are not in agreement with previous data published by Davis and Sowinski [36] who reported closed-ended TNTs in T cells which contained actin filaments but not microtubules.

It is important to underline that if we compared the inhibition of CFSE transfer with the formation of cell conjugated by pharmacological agents, we can observe that a lesser exchange is paired up with an higher percentage of conjugates. In fact, as shown in Fig. 3D the cytochalasin B and PP2 partially affect the cell conjugates formation, whereas latrunculin A strongly impair this phenomenon. The behaviour of nocodazole was further different; as show the scatter gram (Fig. 3D) this treatment lead to a partially inhibition of cell conjugates but at the strongly inhibition in the CFSE transfer. These results suggest that in our experimental condition coexist different types of TNTs and that the microtubule network may propel cytoplasmic material *via* structures like nanotubes as well as favour the cell conjugation.

### Exchange of membranes derived from the endocytic compartment

**Figure 4:**
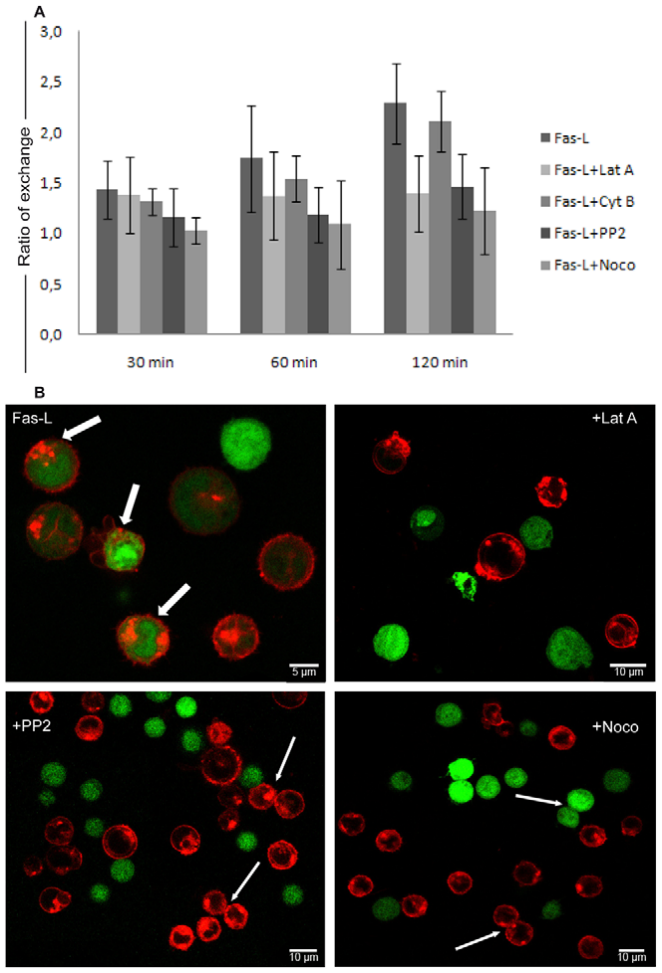
Intercellular exchange of endocytic vesicles DiI-labelled between CD4+ T cells. Bar diagram represent the time-course of DiI transfer, quantified by flow cytometry, after FasL administration and pre-treatment with different pharmacological inhibitors (A). DiI uptake by green cells shows a growing trend over time, which is strongly reduced by Noco treatment, indicating a main involvement of microtubules. The data are a representation of 6 independent experiments and have been expressed as ratio of MFI on treated cells divided by that recorded in untreated cells. Confocal microscopy (B) of cells labelled with CFSE (green) and DiI (red) separately support the cytometric data for both dyes exchange (Figs. 3 and 4A). The image of FasL stimulated cells revealed most “double positive" cells (thick arrows) that are decreased or even absent after pre-treatment with Lat A (latrunculin A) or PP2 or Noco (nocodazole), although some cell conjugates are still present (thin arrows).

To further investigate the possible routes of intercellular communication, we analyzed the transfer of DiI, a dye frequently used as long-term membrane tracer as well as a marker of the endo-lysosomal compartment [14]. The endocytic compartment is the source of membrane vesicles, in particular exosomes, that are released outside cells and can contribute to intercellular exchanges and communication [19]. The results obtained with DiI-stained CD4+ T cells showed that FasL induced the transfer of DiI-stained endocytic material, which was blocked by the actin inhibitor latrunculin, as well as PP2 and nocodazole, but not cytochalasin B (Fig. 4A). Our results thus suggest that Fas stimulation may induce different forms of cell-to-cell communication: an early exchange of cytoplasmic content, which is mainly contributed by TNTs, and a concomitant, and probably more progressive exchange of membranes connected to the endocytic compartment. The cytometric results reported in the statistical histogram are supported by confocal microscopy analyses (Fig. 4B), which evidenced an exchange of dyes (thick arrows) after FasL stimulation. The treatment with pharmacological agents such as latrunculin A, PP2 and nocodazole strongly reduced this exchange but weakly interfered with the formation of cell conjugation (thin arrows). However, in all our experimental conditions each pharmacological agent alone was compared to its control revealing that the inhibitors are not able to induce any type of intercellular communication (data not shown).

### z-VAD effect on intercellular communication

**Figure 5:**
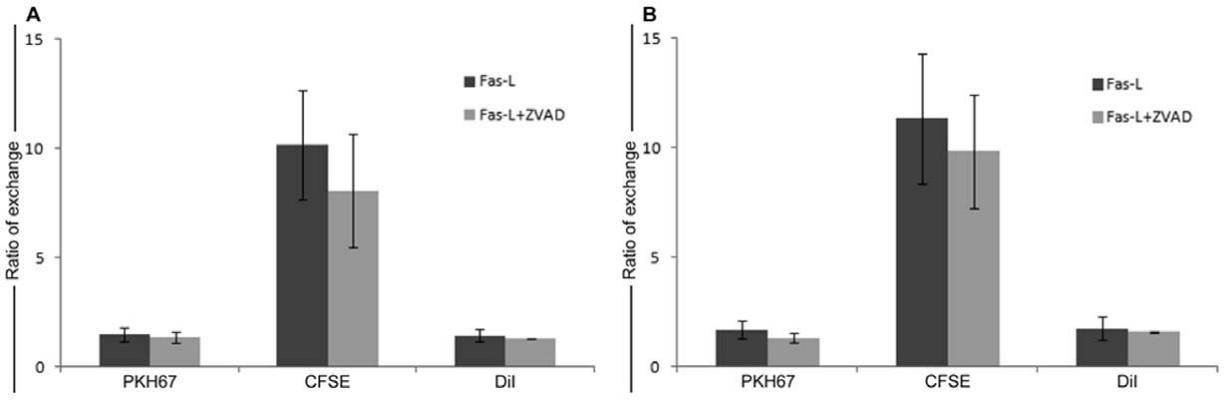
Effect of caspase inhibitor z-VAD on different dyes transfer. Bar diagram represent the exchange of different dyes PKH67, CFSE and DiI evaluated after FasL administration with or without z-VAD pre-treatment. The histograms highlight that z-VAD induces a weak decrease in the CFSE transfer, whereas it is negligible for the other dyes. The reduction of cytoplasmic transfer is more evident after 30 min of FasL administration (A) if compared to 1 h treatment (B). Data presented are mean±SD from n = 3 independent experiments.

FasL is a transmembrane proteins belonging to the tumour necrosis factor (TNF) superfamily that can trigger apoptotic cell death following interaction with its cognate receptor (FasR/CD95). Apoptosis induces by FasL activates caspase-8, providing a direct link between surface death receptors and the caspases. To evaluate the potential involvement of caspases in the intercellular communication, CD4+ T cells were pre-treated with the pan-caspase inhibitor z-VAD at the final concentration of 20 µM. As shown in Fig. 5, z-VAD treatment does not affect the exchange of lypophilic dyes such as PKH and DiI (the measured effect being <10%), while the ratio of CFSE exchange is hardly reduced (<20%). These results suggest that the phenomenon of intercellular communication promoted by FasL is mainly caspase-independent, even if concomitant with the initial activation of caspase-8 in the death signalling cascade.

### Contribution to cell communication by MVs

**Figure 6:**
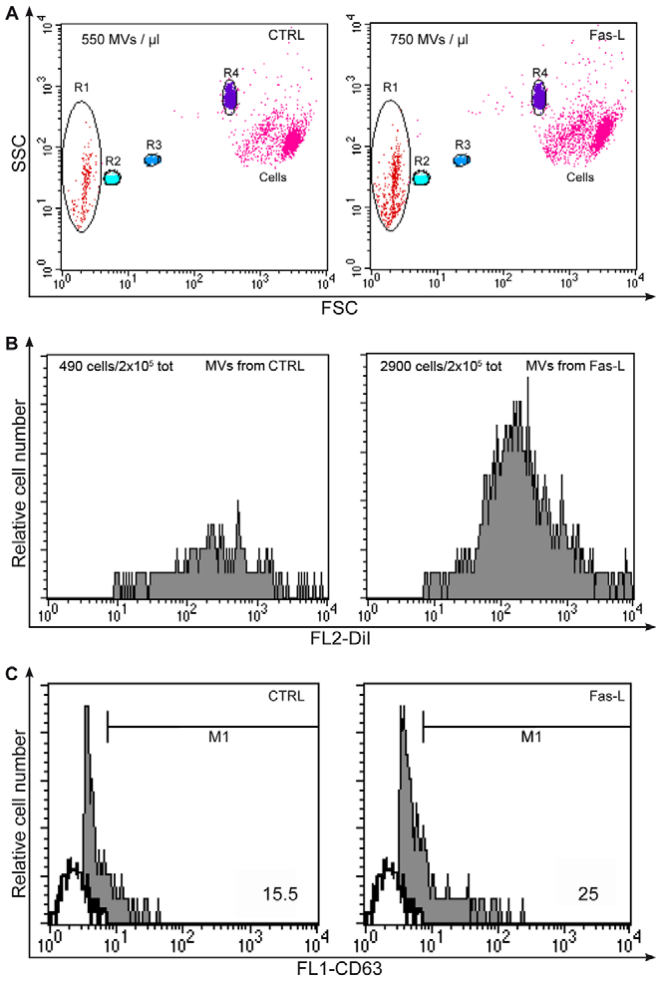
Flow cytometric analysis of FasL effects on MVs release. The release of MVs by CD4+ T cells with (Fas-L) and without (CTRL) FasL administration was evaluated using size beads, identified by R2 (1 µm), R3 (2 µm) and R4 (5,2 µm) regions (A). This gate strategy was performed to define the proper gate for events smaller than 1 µm (R1), which include MVs. FasL treatment performed for 1 h leads to an increase of events within R1 region, demonstrating an higher MVs presence in the medium. Histograms (B) show a higher number of DiI+ events (gated on total cells) in CD4+ T cells after the addition of DiI+ MVs released by FasL treated samples, attesting a major uptake in these ones if compared to acquired DiI+ MVs from untreated control cells. (C), Shown are histograms of CD63-FITC staining on R1 gated MVs. CD63 expression (%) is derived from overlay of FL1 empty channel (white histograms) and CD63 filled channel (grey histograms).

The results obtained with the DiI-stained CD4+ T cells (Fig. 4) suggested that some of the observed exchanges stimulated by FasL and sensitive to various pharmacological inhibitors were contributed by the release of membrane vesicles (particles) and/or exosomes. Therefore, we investigated whether Fas stimulation induced the release of membrane vesicles and exosomes from T cells. In previous study of time-lapse imaging of Jurkat T cells, we had observed a sharp increase in the release of membrane vesicles 30–40 min after Fas stimulation (cf. Fig. 1 in Ref. [28] and M. Degli Esposti, unpublished results). The amount of released membrane vesicles has been measured by their protein content after separation by ultracentrifugation (see methods). This increased from 0.2 to 1.8 µg of protein for 10^7^ cells. More recently, we applied flow cytometry to study the release of membrane vesicles from primary T cells [32]. The flow cytometry approach consists of mixing untreated and Fas treated CD4+ T cells with beads of defined size (Ø 1 µm, 2 µm, 5.2 µm) to obtain a size calibration of small particles (the majority falling within gate R1 in Fig. 6A) detected outside the scatter area of intact cells. Of note, activated T cells release MVs, thus explaining the substantial amount detect in control cells (Fig. 6A); however, we observed that FasL treatment increased this release significantly (numbers of R1 gated events in Fig. 6A, 1.4 fold of increase). Furthermore, after the co-culture between unstained CD4+ T cells and DiI+ MVs, we have detected the presence of DiI+ events in the cell scatter area, suggesting the uptake of MVs by CD4+ lymphocytes (Fig. 6B). We found a higher uptake (about 6 fold of increase) for MVs obtained from FasL treated cells, if compared to MVs released by untreated ones. In addition, membrane vesicles separated by ultracentrifugation were investigated for their expression of the endo-lysosomal marker CD63 [37]. In the cytometric data shown in Fig. 6C, the white histogram correspond to the negative control, whereas the light grey histogram show the MFI (mean fluorescence intensity) of positive events for CD63 expression (15.5% for MVs from controls *vs* 25% for MVs from samples 1 h-treated with Fas).

### Evaluation of the release of endogenous FasL

**Figure 7:**
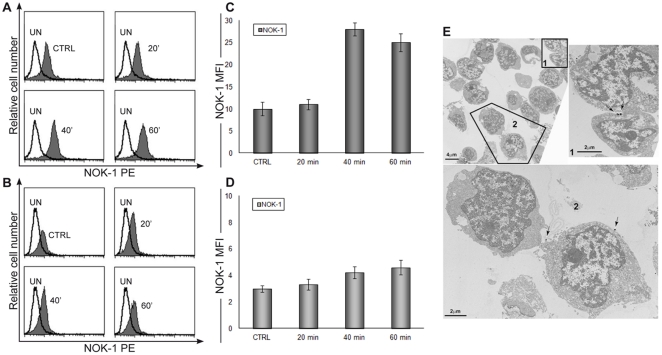
Detection of intracytoplasmic and surface FasL by a mAb anti-FasL NOK-1. Flow cytometric analysis of intracytoplasmic (A) and surface (B) NOK-1 PE stain of CD4+ T cells at different time points of CH-11 treatment (20′, 40′ and 60′). The treated histograms are compared to untreated (CTRL) and unstained (UN) samples. The statistical histograms (C,D), relating to previous cytometric data, are the mean±SD from 3 separate experiments. The intracytoplasmic NOK-1 PE stain (A,C) show a strongly increase after 40 min of CH-11 treatment, if compared to NOK-1 surface detection (B,D), where the MFI values are similar to all time points. The trend of the intracytoplasmic detection indicated a FasL mobilization. Immunoelectron microscopy (E) of FasL distribution in cell couplings by NOK-1 stain. Magnified views (1,2) demonstrate FasL labelling in the surface and contact areas, indicated by the arrows. The panel shows a representative image of immunogold labelling that was obtained as detailed in the Materials and Methods section.

Having established that Fas stimulation enhanced also the release of membrane vesicles in primary T cells, we have then investigated the cytoplasmic and surface expression of endogenous FasL using the specific antibody NOK-1 (Fig. 7), before and after stimulation of the Fas receptor with the agonist antibody CH-11. Previous studies have reported the association of membrane-bound FasL to MVs and exosomes which can be released from ‘killer’ cells even within an homotypic population [38]. Our results indicate an abrupt increase in the levels of endogenous FasL within cells (Figs. 7A and C). Clearly, part of the FasL in the cell was also associated with exosomes, as observed by immunoelectron microscopy (Fig. 7E). Interestingly patches of FasL could be detected on the surface of conjugated CD4+ T cells (Fig. 7E), in accordance with previous results obtained in transformed T cells [28].

## Discussion

In this study we provide further evidence supporting novel aspects of Fas signalling, beyond its well documented intracellular activity in the context of apoptosis. Fas is a cell-surface receptor belonging to the tumour necrosis factor receptor (TNF-R) superfamily. Once triggered by its cognate ligand (FasL), it initiates a cascade of events within the cells that ultimately culminates in their death. This process involves the formation of death-inducing signalling complex, consisting mainly of the Fas-associated death domain and the caspase 8 and caspase 10 proteins. Our results additionally show that Fas triggering with FasL also stimulates intercellular forms of communication, that we evaluated in depth by flow cytometry and confocal microscopy. To date, several reports have documented that similar exchanges occur in different cells of the lymphoid lineage such as T and B lymphocytes, NK and dendritic cells after establishing IS. Formation of an IS by T, B or NK cells is associated with an intercellular transfer of some membrane fragments from cells in intimate contact with each other [31]. This capture is thought to require effector cell activation by surface recognition of stimulatory ligands.

Now we can suggest that FasL is among the physiological ligands that are capable of stimulating T cells to produce contact-mediated membrane exchanges, although it is important to underline that this form of transfer isn't the only one to be promoted by this type of ligand.

The exchange of membrane fragments, referred to as trogocytosis, was documented for the first time by Joly and Hudrisier [3] in the immunological system. Trogocytosis is an active transfer phenomenon, which is triggered specifically by antigen receptor signalling. To remain within T cells, Hwang et al. [6] have shown TCR-independent membrane transfer between APC and pre-activated T cells, involving engagement of CD28 and its ligand B7-1/2, thereby suggesting the existence of an alternative antigen-independent pathways of trogocytosis. Indeed, it has been recently demonstrated that trogocytosis can be triggered by specific surface receptors, either individually or in combination, without the need of CD28/TCR activation [8]. In line with this finding, Waschbish et al. [39] demonstrated that the transfer of membrane fragments during myoblast-T cell crosstalk did not require TCR engagement.

Our results clearly demonstrated the formation of cell conjugates in early time-frame of Fas stimulation, a pre-requisite for cell communication that can occur also *via* TNTs and MVs release. In fact, the analysis performed in our experimental conditions by PKH26 and PKH67 indicated a poor transfer of plasma membrane fragments, whereas the stain with CFSE and the higher transfer of this cytoplasmic dye led us to think that the interaction of Fas and its cognate FasL was able to induce the cytoplasmic transfer.

In 2004, a new way of cell-to-cell communication was discovered based on the formation of thin membrane channels connecting mammalian cells in culture [9], [14]. These channels, called TNTs, were shown to mediate membrane continuity between PC12 (and other cell types) to facilitate the intercellular transfer of vesicles of endocytic origin as well as, on limited scale, of membrane components and cytoplasmic molecules. Regardless of the possible distinction between membrane bridges and nanotubes, or different nanotubular structures, it remains unclear what kind of components can be transferred *via* such structures, although the discovery of novel TNTs cargo is a rapidly evolving field. The characterization of nanotube is still controversial, but a recent report [40] underline that actin polymerization is essential for the development of TNTs.

To confirm our findings that TNTs are involved in the cytoplasmic exchange, suggested by a remarkable CFSE transfer, we pre-treated CD4+ T cells with different chemical agents. The significant inhibition obtained with latrunculin and nocodazole suggest the importance of the actin and microtubules in the TNTs formation in our experimental model. We are mindful that flow cytometry cannot properly evaluate the presence of TNTs in primary T cells, which require accurate morphological analysis for proper detection [28], [32], [41]. For this reason, we have paired up the flow cytometry studies with some confocal microscopy data (Fig. 3) that clearly evidenced the presence of TNTs and the exchange of dyes. The presence of TNTs is a very intriguing structure in a “classical apoptotic model" as Fas-FasL for different reasons. First because albeit a growing numbers of studies have detail the mechanisms of programmed cell death, in all such studies cells are considered as single entities that are differently affected by various triggers. Our data additionally indicate that during the apoptotic pathway there is an early time-frame in which cells exchange different materials, such as proteins or lipids that can propagate and expand the signal to die among activated T cells. Moreover, our results are in alignment with previous reports demonstrating an induction of intercellular communication by cellular stress, for example after treatment of astrocytes with hydrogen peroxide [27]. Likewise, expression and formation of other intercellular channels such as gap junctional communication was reported to be induced after activation of microglia and monocyte/macrophages lineage by various compounds and stress stimuli [40]. These and our data indicate that tube-genesis might constitute a cellular strategy to facilitate cell-to-cell communication in response to stress condition. The significant CFSE shift and DiI staining, as endocytic compartment dye [14], suggested to us that FasL stimulation of CD4+ T cells could also promotes other routes for cytoplasmic transfer, such as MVs release.

MVs are increasingly recognized as mediators of intercellular communication due to their capacity to merge with and transfer a repertoire of bioactive molecular content (cargo) to recipient cells. Different biological circumstances under which formation of MVs has been observed reflected the diversity of their biogenesis, structure and function. Thus, cellular activation, transformation, stress, or programmed cell death are associated with a different output and nature of vesicular structures [42]. It is known that MVs are heterogeneous and this has led to the usage of multiple names for their designation under different experimental settings [43]. Our data suggest that the treatment induces the release of MVs and in this heterogeneous pool we identified both Anx-V positivity (data not shown) and CD63 positive events, qualifying these last events as exosomes. It is known that the release of MVs have an important role in the physiological and pathological conditions; in fact the cargo of MVs includes growth factors and their receptors, proteases, adhesion molecules, signalling molecules, as well as DNA, mRNA and micro-RNA sequences. Obtained results highlight a new aspect for the MVs in the apoptotic pathway where their cargo with bioactive molecules can modify the characteristic of the recipient cells. These and our previously findings [32] demonstrated that the MVs amplify the apoptotic phenomenon; in fact the cells during dead could release MVs containing death signals able to interfere with neighbour cells. Our results suggest that FasL treatment induces a transcellular apoptosis, phenomenon recently described by Sarkar and co-workers [44]. CD4+ T cells triggered with FasL are able to release MVs (exosomes/shedding vesicles) that could direct the cells to apoptotic pathway. In fact, CD4+ T cells co-cultured with MVs, isolated after FasL treatment, seems to induce a mitochondrial impairment (further investigation are in progress). On the other hand, these findings are reinforced and supported by our observation that Fas stimulation induces a mobilization of FasL by the secretory lysosomes within 1 h of treatment, with a peak after 40 min (Fig. 7). In fact, growing numbers of evidences reported that a tight regulation of FasL surface expression is essential to prevent non-specific killing by T cells [45]. Whereas the intracytoplasmic stain with NOK-1 displays a significant shift after 40 min, the surface staining shows weak changes. This behaviour led us to believe that FasL could be mobilized and subsequently released in the environment as soluble form and/or within MVs.

To conclude our data suggest that Fas signalling, a well-known pathway of apoptosis, promotes also intercellular communication which simultaneously occur during apoptotic phenomenon, essential for development, homeostasis and maintenance of multicellular organisms.

## Materials and Methods

### Ethics Statement

CD4+ T cells were isolated from buffy coats of fully anonymized donors obtained from the Transfusion Centre of Urbino Hospital. No specific approval from an institutional review board is required for the use of buffy coats for the following reasons: (1) no personal patient information is made available, (2) buffy coats cannot be used for treatment of patients and are waste products for the blood transfusion centre and (3) blood donors were verbally informed that parts of the donation that cannot be used for patient treatment may be used for scientific research.

### Cell culture and treatments

PBMC were isolated from buffy coats of adult healthy donors, obtained anonymously. Blood was diluted two-fold with PBS containing 2 mM EDTA, and then stratified on Ficoll-Paque tubes to isolate PBMC by centrifugation. CD4+ T cells were purified from PBMC by negative selection using the MACS system (Milteny Biotec, Auburn, CA). T cells were then treated with PHA and rIL-2 (100 U/ml, Prepotech, London, UK) for different times of post-incubations [46]. CD4+ T cells were cultured in RPMI 1640 supplemented with 10% FCS (fetal calf serum), 2 mM glutamine, 100 U/ml penicillin and 100 µg/ml streptomycin. CD4+ T cells were treated with FasL (Apothech/Alexis Lausanne, Switzerland) at the final concentration of 0.5 µg/ml for 2 h. Cell viability was evaluated by Trypan blue exclusion test and flow cytometry (FC).

### Cell labelling

CD4+ T cells were split in two groups, which were stained either green, with the lypophilic dye PKH67 and the cytosolic stain CFDA-SE (CFSE) and red, with the lipophilic dye PKH26 and DiIC_18_ (DiI). For PKH staining, 10×10^6^ cells were washed in PBS with 2 mM EDTA, then the pellet was resuspended in 500 µl of diluent C (Sigma Aldrich). Both PKH were diluted to a final concentration of 2–4 µM with the same diluent, rapidly added to cells and incubated for 5 min at room temperature, with occasional gentle agitation. Then cells were supplemented with 2 ml of FCS, incubated at room temperature for 1 min and washed three times in complete culture medium. For staining with CFSE, cells were incubated for 10 min at 37°C with 1 µM CFSE, followed by quenching of excess staining with cold FCS and two washings in complete medium [30]. For DiI staining, the probe was diluted in 250 µl diluent C, rapidly added to the cell suspension at a final concentration of 40 µg/ml and incubated at room temperature for 5 min with occasional agitation. Reaction was stopped with the addition of cold FCS and cells were washed three times in complete culture medium [29]. After labelling, cell samples were treated for 30 min at 37°C with: latrunculin A (2.5 µg/ml), cytochalasin B (10 µM), PP2 (10 µM) nocodazole (20 µM) and z-VAD (20 µM).

### Evaluation of passive diffusion

2×10^5^ CD4+ T unstained cells were mixed with 2×10^5^ fluorescently labelled T cells in 96-well plates in 100 µl of final volume and incubated at 37°C for 30, 60 and 120 min. Basal levels of membrane exchange, measured from the extent of dye dilution in the mixed population, were assessed by FC by comparing the mean fluorescence intensity (MFI) of the unstained cells before and after incubation with the stained cells. The basal exchange of each probe was calculated as a percentages using the formula: (MFI of unstained cells after diffusion – MFI of unstained cells)/(MFI of stained cells – MFI of unstained cells)×100 [29].

### Fas-mediated intercellular exchange

After differential labelling, cells stained red or green were mixed at 1∶1 ratio and then stimulated with FasL at the final concentration of 0.5 µg/ml for 2 h. To promote cell conjugate formation, a short centrifugation step was included (30 sec at 1,100 rpm) and then left at 37°C for different times of subsequent incubation [47]. The exchange of membrane or cytosolic elements was determined from the acquisition of green fluorescence by red stained cells or reciprocally of red fluorescence by green stained cells. Quantification of the exchange at each time of incubation was calculated as a ratio of the signal on stimulated cells divided by that recorded in control untreated cells using the formula: green MFI of red cells at 30, 60 or 120 min/green MFI of red cells at time 0 [29]. Data were acquired and analyzed with FACScan flow cytometry (Becton Dickinson, Pablo Alto, CA) using the CellQuest software.

### Detection of programmed cell death

FasL-induced apoptosis was assessed using Anx-V FITC apoptosis detection kit (Immunostep). After an incubation step at room temperature samples were processed for the flow cytometric analysis. The CaspGLOW Fluorescein Multi-Caspase Staining Kit (BioVision, CA) was used to evaluate the total activity of caspase enzymes. For both tests we followed manufacturer's instructions [48]. Samples acquisition was performed at 30 min and 120 min after FasL administration by FACScan flow cytometer equipped with CellQuest software (BD Biosciences, Milan, Italy); data were expressed as percentage of positivity.

### Microvesicles (MVs) isolation and characterization

MVs were purified by differential centrifugation by CD4+ T cells stained with DiI. Conditioned or control medium from CD4+ T cells was centrifugated for 15 min at 1,000 g to eliminate cell contamination. Supernatants were further centrifugated for 20 min at 12,000 g and subsequently for 20 min at 18,000–20,000 g. The resulting supernatants were filtered through a 0.22 µm filter and then MVs were pelleted by ultracentrifugation at 110,000 g for 70 min. The MVs protein quantification was performed by Bradford assay [49]. After the MVs isolation from the control and conditioned medium, CD4+ T cells were seeded in the cell culture plates at the final concentration of 2.5×10^6^/ml with the MVs (1–5 µg) within 1 h of co-culture. The MVs were washed and resuspended in PBS and then stained with an anti-CD63 FITC (clone TEA3/18). The cytometric analyses were performed by gating events smaller than 1 µm. Size beads (Ø 1–2 µm Polysciences Invitrogen, Carlsbad, CA and Ø 5.2 µm DakoCytoCount beads) were used to establish the proper gate for events smaller than 1 µm, which include MVs, and to obtain single platform absolute counts [50].

### Surface and intracellular staining of FasL

CD4+ T cells were seeded in 96-well plates at the concentration of 1×10^6^/ml and treated with mAb anti-Fas CH-11 at the final concentration of 0.4 µg/ml for 20, 30, 40 and 60 min. The release and the intracytoplasmic detection of FasL were evaluated by a mAb anti-FasL NOK-1 PE. Membrane surface expression of FasL was detected by NOK-1 MFI values. For the intracytoplasmic detection CD4+ T cells were washed in PBS and resuspended in 250 µl of Cytofix reagent and incubated at 4°C for 30 min. Then, the cells were washed in the washing buffer and resuspended in 250 µl of Cytoperm reagent. An anti-NOK-1 PE conjugated was added and the cells were incubated at 4°C for 30 min. Staining with PE conjugated isotype control and autofluorescence evaluation were performed to assess unspecific fluorescence.

### Immunocytochemistry and fluorescence microscopy

Cells were washed and resuspended in modified Ringer buffer (RB, containing 145 mM NaCl, 4.5 mM KCl, 2 mM MgCl_2_, 1 mM CaCl_2_, 5 mM K-Hepes, pH 7.4, and 10 mM glucose), loaded with fluorescent probes for different times, washed and incubated at 4–6×10^6^/ml with FasL before plating into cover slips coated with poly-lysine [51]. After adhering for 15–20 min at 37°C, cells were transferred on ice and then washed with cold PBS before fixation with 4% (w/v) paraformaldehyde. We routinely imaged cells with DeltaVision RT (software Rx. 3.4.3, Applied Precision) at 20°C using an automated Olympus IX71 microscope with oil-immersed objectives [28]. Images from stacks of 25–35 sections of 0.2 µm were deconvolved for 10 cycles and then projected along the z-plane. Furthermore, we used a Leica TCS SP5 II confocal microscope with oil-immersed objectives. In this case, Z projections were generated using ImageJ software.

### Electron microscopy studies

Cells were treated with an anti-Fas CH11 and attached to glass coverslip pre-treated with poly-lysine; staining followed by a PBS wash before fixation with 3.8% acrolein (TAAB, Aldermaston, UK) plus 2% paraformaldehyde in 0.1 M phosphate buffer (pH 7.4) for 1 h at room temperature [28]. Coverslips were then postfixed in 2% osmium tetroxide for 1 h and subsequently dehydrated by a series of ethanol and propylene oxide washes, prior to embedding in Epon 812 between sheets of Aclar plastic (Agar Scientific, Stansted, UK). Before preparing the final Epon block, coverslip glass was removed with hydrofluoric acid. Ultrathin sections were cut with a diamond knife (Diatome), collected on copper mesh grids and counterstained with uranyl acetate plus lead citrate.

Immunogold labelling of FasL was carried out after incubating the pellets of Fas-stimulated T cells for 48 h at 4°C in Tris-buffered saline containing the NOK-1 monoclonal at 1∶50 dilution in 0.1% BSA. Subsequently, the primary antibody was detected by using the pre-embedding immunogold-silver method and a rabbit anti-mouse IgG conjugated with 10 nm colloidal gold (British Biocell International, Cardiff, UK) for 2 h and then rinsed in BSA/gelatin/PBS. The bound gold particles were secured by placing the sections in 2% glutaraldehyde in 0.01 M PBS for 10 min. Pellets were then post-fixed in 2% osmium tetroxide for 1 h and subsequently dehydrated by a series of ethanol and propylene oxide washes prior to embedding in Epon 812 (Agar Scientific, Stansted, UK). Images were obtained with a Philips Tecnai electron microscope and composite electron microscope figures were generated using Adobe Photoshop CS. This work was undertaken in collaboration with Dr. J. Rodriguez-Arellano at the University of Manchester.

### Statistical analysis

Data are shown as mean ± standard deviation of at least three independent experiments performed in duplicate. The Student *t* test was used to perform the statistical analysis and values of p<0.05 were considered to be significant.

## Supporting Information

Figure S1 **Detection of apoptotic parameters.** Caspases and Anx-V positivity were quantified by flow cytometry after 30 min and 120 min of Fas-L administration. Percentage values were obtained subtracting positive cells from the same samples without FasL. The bar diagram shows that at 30 min a few cells were already Anx-V+ and Caspases+, increasing widely after 120 min of Fas-L addition. These data highlight that apoptotic pathway starts after FasL stimulation, suggesting that in our experimental conditions, Fas/FasL interaction concomitantly promotes apoptosis and enhances intercellular communication.
(TIF)
