# Supplementary figures and images for: Fas Signalling Promotes Intercellular Communication in T Cells
Source: PLoS One. 2012 Apr 25;7(4):e35766. doi: 10.1371/journal.pone.0035766 (PMC3338457; doi:10.1371/journal.pone.0035766)

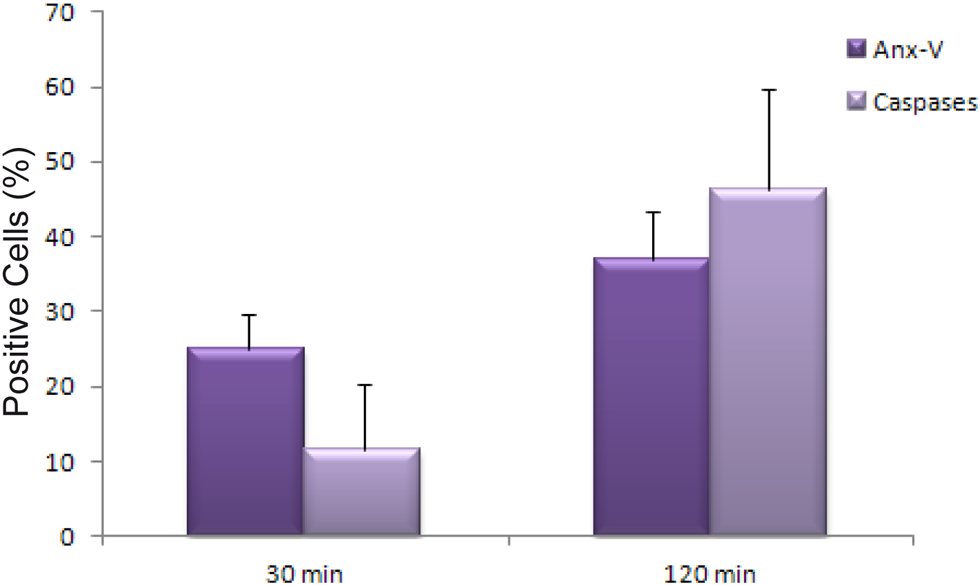

Supplement: Figure S1 — Detection of apoptotic parameters. Caspases and Anx-V positivity were quantified by flow cytometry after 30 min and 120 min of Fas-L administration. Percentage values were obtained subtracting positive cells from the same samples without FasL. The bar diagram shows that at 30 min a few cells were already Anx-V+ and Caspases+, increasing widely after 120 min of Fas-L addition. These data highlight that apoptotic pathway starts after FasL stimulation, suggesting that in our experimental conditions, Fas/FasL interaction concomitantly promotes apoptosis and enhances intercellular communication. (TIF) [file pone.0035766.s001.tif]
